# Normal spermatogenesis in *Fank1* (fibronectin type 3 and ankyrin repeat domains 1) mutant mice

**DOI:** 10.7717/peerj.6827

**Published:** 2019-04-24

**Authors:** Jintao Zhang, Xin Zhang, Yue Zhang, Wentao Zeng, Shuqin Zhao, Mingxi Liu

**Affiliations:** 1 Department of Histology and Embryology, Nanjing Medical University, Nanjing, China; 2 Animal Core Facility of Nanjing Medical University, Nanjing, China

**Keywords:** Fank1, Male infertility, Gene knockout, Spermatogenesis

## Abstract

**Background:**

The fibronectin type 3 and ankyrin repeat domains 1 gene, *Fank1*, is an ancient, evolutionarily conserved gene present in vertebrates. Short-hairpin RNA (shRNA)-based knockdown transgenic mice have oligospermia caused by an increase in apoptotic germ cells. In this study, we investigated the in vivo function of *Fank1*.

**Methods:**

In this study, we generated *Fank1*-knockout mice using the CRISPR/Cas9 system. We then investigated the phenotype and in vivo function of *Fank1*. Testes and epididymis tissues were analyzed by histological and immunofluorescence staining. Apoptotic cells were analyzed in terminal deoxynucleotidyl transferase dUTP nick end-labeling assays. Fertility and sperm counts were also evaluated. The GTEx database were used to assess gene expression quantitative trait loci and mRNA expression of candidate genes and genes neighboring single nucleotide polymorphisms was analyzed by quantitative RT-PCR.

**Results:**

In contrast to the *Fank1*-knockdown model, no significant changes in epididymal sperm content and the number of apoptotic cells were observed in *Fank1*−/− homozygotes. In addition, a different pattern of Dusp1, Klk1b21 and Klk1b27 mRNA expression was detected in *Fank1*-knockout testis. These results reveal differences in the molecular changes between *Fank1*-knockdown mice and *Fank1*-knockout mice and provide a basic resource for population genetics studies.

## Introduction

Genetic studies are widely used for identification of susceptibility loci in human disease (Johnson & O’Donnell, 2009). Mouse models of gene editing are indispensable for investigations of gene function in vivo. However, the development of genetic research is restricted by the lack of progress in our understanding of gene function. Thus, large-scale knockout programs have been initiated to mutate all protein-encoding genes in the mouse (Collins, Rossant & Wurst, 2007; Skarnes et al., 2011). The CRISPR/Cas9 system has been used to target genomic loci in mammalian studies (Li et al., 2013; Mali, Esvelt & Church, 2013; Shen et al., 2013; Wang et al., 2013), and gene knockout mice have become more commonly used in genetic studies in mice. To date, 1,503 human diseases with one or more mouse models have been recorded in the Mouse Genome Informatics database (Smith et al., 2018).

The fibronectin type 3 and ankyrin repeat domains 1 gene (*Fank1*) is an ancient, evolutionarily conserved gene present in vertebrates and expressed from the meiosis phase to the haploid phase of spermatogenesis in the testis (Zheng, Zheng & Yan, 2007). As a DNA binding protein, FANK1 recognizes the DNA sequence AAAAAG, and is implicated as a transcription factor during spermatogenesis (Dong et al., 2014). In a study of short-hairpin RNA (shRNA)-based knockdown transgenic mouse model, a reduction in sperm number and an increase in apoptotic germ cells were observed (Dong et al., 2014).

In recent years, gene editing mouse models have played an indispensable role in elucidating gene function in vivo. A number of studies have revealed phenotypic differences between knockout (i.e., mutants) and knockdown (i.e., RNA interference) models (El-Brolosy & Stainier, 2017). These phenotypic differences could be caused by gene expression compensation in mutants or off-target effects of the knockdown reagents (El-Brolosy & Stainier, 2017). Both models have distinct advantages and limitations for the elucidation of gene function. Gene knockdown is usually achieved with exogenous reagents, for example morpholino, siRNA, shRNA and antisense morpholino oligos (El-Brolosy & Stainier, 2017; Nasevicius & Ekker, 2000). These reagents usually induced sequence-dependent repression of the target gene in several different ways, such as mRNA degradation, translational inhibition or transcriptional inhibition, which do not involve genomic mutation. However, gene knockouts are generated directly by genome editing and may be a better model of human genetic mutations.

Thus, in this study, we have generated a *Fank1*-mutant model using the CRISPR/Cas9 system to investigate the phenotype and in vivo function of *Fank1*.

## Materials and Methods

### Gene expression quantitative trait loci analysis

The publicly available RNA-seq and genotyping data of human samples from the Genotype-Tissue Expression project (GTEx, http://commonfund.nih.gov/GTEx/index) were used to assess gene expression quantitative trait loci (eQTL) for mRNA expression of candidate genes and genes neighboring single nucleotide polymorphisms (SNP). Statistical analysis was performed using The GTEx Consortium (2013).

### Generation of *Fank1*-knockout mice by CRISPR/Cas9

The mice were maintained and used in experiments according to the guidelines of the Institutional Animal Care and Use Committee of Nanjing Medical University (China). The animal use protocol has been reviewed and approved by the Animal Ethical and Welfare Committee (Approval No. IACUC-1601117 and IACUC-1811001). Cas9 mRNA and single guide RNAs (sgRNAs) were produced and purified as previously described (Zhang et al., 2017). In brief, the Cas9 plasmid (Addgene, Watertown, MA, USA) was linearized by restriction enzyme digestion with *Age*I and then purified using a MinElute PCR Purification Kit (Qiagen, Duesseldorf, Germany). Cas9 mRNA was produced by in vitro transcription using a mMESSAGE mMACHINE T7 Ultra Kit (Ambion, Austin, TX, USA) and purified using a RNeasy Mini Kit (Qiagen, Duesseldorf, Germany) according to the manufacturer’s instructions. The sgRNAs were designed on the basis of exon2 of *Fank1*. The target sequence of sgRNA was 5′-GTGGCTTCGGTTCTCCATTGAGG-3′ and 5′-GTCACCTTGCCCACAACAGGAGG-3′, respectively. The sgRNA plasmid was linearized with *Dra*I and then purified using a MinElute PCR Purification Kit (Qiagen, Duesseldorf, Germany). sgRNA was produced using the MEGA shortscript Kit (Ambion, Austin, TX, USA) and purified using the MEGA clear Kit (Ambion, Austin, TX, USA) according to the manufacturer’s instructions. Cas9 mRNA and sgRNA were injected into mouse zygotes obtained by mating of wild-type C57BL/6 males with C57BL/6 superovulated females.

### Genotyping

Edited founders of *Fank1* were identified by PCR amplification (Rapid Taq master mix, Vazyme) with primers (Primer F 5′-GGTCCACAGTTGTTGTTGCT-3′ and R 5′-ATTCCAAGAGTCCATCGGTTCA-3′) and subcloned into pMD19-T (TaKaRa) followed by standard Sanger sequencing. The length of the corresponding wild-type and mutant alleles were 403 and 333 bp, respectively. The selected founder was crossed with wild-type C57BL/6J to eliminate possible unwanted off-targets and to generate pure heterozygous mice. *Fank1*−/− mice were resequenced by Sanger sequencing and the results were plotted using SnapGene (version 1.1.3). Genotyping was performed by agarose gel electrophoresis analysis of PCR products produced from DNA isolated from tail biopsy specimens.

### Western blot analysis

Testicular protein extracts were prepared using lysis buffer [8 M urea, 75 mM NaCl, 50 mM Tris-HCl, PH8.2] containing 1× cOmplete™ EDTA-free Protease Inhibitor Cocktail (Roche, Basel, Switzerland). The proteins were separated by SDS-PAGE and transferred onto a polyvinylidene difluoride membrane. The membranes were blocked with 5% non-fat milk in TBS-T (20 mM Tris, 150 mM NaCl, 0.1% Tween 20) for 2 h at room temperature and incubated overnight at 4 °C with the following primary detection antibodies: anti-FANK1 (sc-398026; Santa Cruz Biotechnology, Santa Cruz, CA, USA) at a dilution of 1:1,000 and anti-β-actin (ac026, ABclonal, Wuhan, China) at a dilution of 1:10,000. The membranes were washed with TBS-T buffer three times and incubated at room temperature for 2 h with secondary detection antibodies at a dilution of 1:2,000. The signals from the detected proteins were visualized using High-sig ECL Western Blotting Substrate (Tanon, Shanghai, China).

### Histological analysis

Mouse testes or epididymis were collected from at least three mice for each genotype. The tissues were fixed in modified Davidson’s fluid for up to 24 h and stored in 70% ethanol. The samples were then dehydrated through a graded ethanol series and embedded in paraffin. Tissue sections (thickness five mm) were prepared and mounted on glass slides. After deparaffinization, slides were stained with periodic acid Schiff for histological analysis. Apoptotic cells in testis were detected using the terminal deoxynucleotidyl transferase dUTP nick end-labeling (TUNEL) assay (Vazyme, Nanjing, China) according to the manufacturer’s instructions.

### Immunofluorescence analysis

Testis sections were deparaffinized, rehydrated and boiled for 15 min in sodium citrate buffer for antigen retrieval. Sections were blocked in antibody dilution buffer (5% bovine serum albumin in phosphate-buffered saline (PBS) 137 mM NaCl; 2.7 mM KCl; 10 mM Na_2_HPO_4_ and 2 mM KH_2_PO_4_) for 2 h at room temperature, followed by an overnight incubation at 4 °C with primary antibodies (list in Table S2). Three washes with PBST (0.05% Tween 20 in PBS) were performed prior to incubation with secondary antibody (list in Table S2) for 2 h at room temperature. Finally, sections were incubated with Hoechst 33342 (Invitrogen, Carlsbad, CA, USA) for 5 min and then mounted. Images were captured using an LSM800 confocal microscope (Carl Zeiss AG, Jena, Germany).

### Fertility test

Adult males of each genotype were subjected to fertility tests. Each male was mated with three wild-type C57BL/6 females, and the vaginal plug was checked every morning. The dates of birth and number of pups in each litter were recorded.

### Computer-assisted sperm analysis

Mature sperm were obtained by making small incisions throughout the cauda epididymis, followed by extrusion and suspension in human tubal fluid culture medium (In Vitro Care, Frederick, MD, USA). Sperm samples (10 μl) were used for computer-assisted semen analysis (Hamilton-Thorne Research, Inc., Beverly, MA, USA). Motile sperm number, progressive sperm number and sperm concentration for the experimental and control groups were measured and analyzed.

### Quantitative RT-PCR assay

Total RNA was extracted from the samples using TRIzol reagent (Invitrogen, Carlsbad, CA, USA). The concentration and purity of RNA were determined using a NanoDrop 2000C (Thermo, Waltham, MA, USA) absorbance at 260/280 nm. Total RNA (one μg) was reverse transcribed using a HiScript II Q RT SuperMix (Vazyme, R222, Nanjing, China) according to the manufacturer’s instructions. The cDNA (dilution 1:4) was then analyzed by quantitative RT-PCR in a typical reaction of 20 μl containing 250 nmol/l of forward and reverse primers, one μl cDNA and AceQ qPCR SYBR Green Master Mix (Vazyme, R222, Nanjing, China). The reaction was initiated by preheating at 50 °C for 2 min, followed by 95 °C for 5 min and 40 amplification cycles of 10 s denaturation at 95 °C and 30 s annealing and extension at 60 °C. Gene expression was normalized to 18 s within the log phase of the amplification curve. The primer sequences are listed in Supplementary Table S3 and Fig. S1.

### Statistical analysis

All experiments were repeated at least three times. The differences between treatment and control groups were analyzed using one-way ANOVA or unpaired two-tailed *t*-tests. *P*-values < 0.05 were considered to indicate statistical significance. All data represent the mean ± the standard error of the mean. Analyses were performed using the Microsoft Excel and GraphPad Prism 6.0.

## Results

### Association of 54 SNPs with Fank1 expression in humans

**Figure 1:**
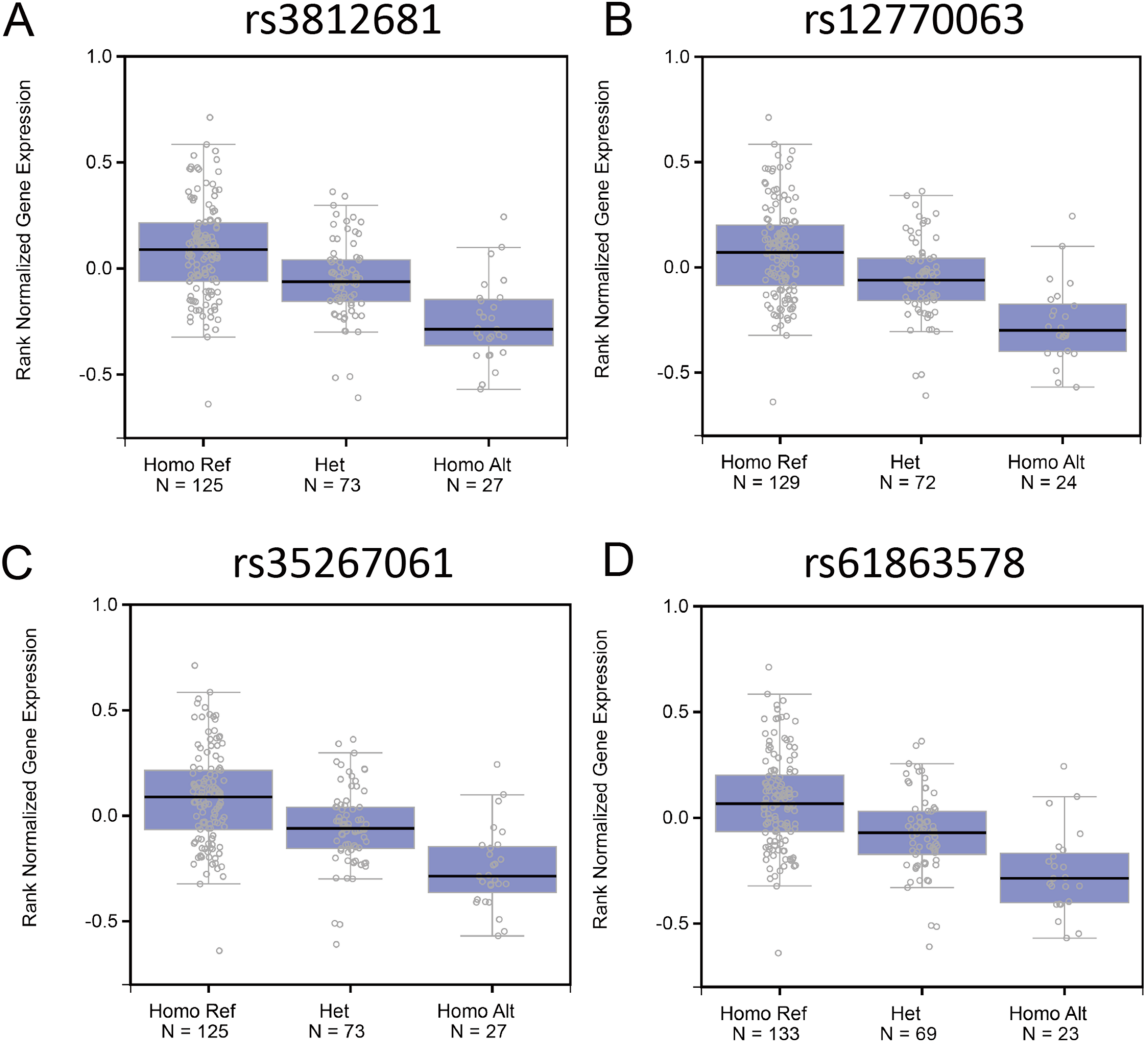
The association of the genotypes of the SNPs with *Fank1* mRNA expression. eQTL analysis of *Fank1* mRNA expression level for genotypes Homo Ref, Het and Homo Alt at (A) rs3812681, (B) rs12770063, (C) rs35267061 and (D) rs61863578.

Genome variants including common SNPs contribute to gene expression changes and are associated with human disease. To investigate the association of the genotypes of the SNPs with *Fank1* mRNA expression, eQTL of *Fank1* and relative SNPs. The eQTL data revealed lower *Fank1* mRNA expression levels in testicular subsets with homozygous genotypes of 54 SNPs compared with that of the homozygous reference (Table S1; Fig. 1). This result revealed the diversity of testicular *Fank1* expression levels in the population. Therefore, it is particularly important to study the phenotype of *Fank1*-deficient mice.

### Fank1−/− mice are fertile and have normal spermatogenesis

**Figure 2:**
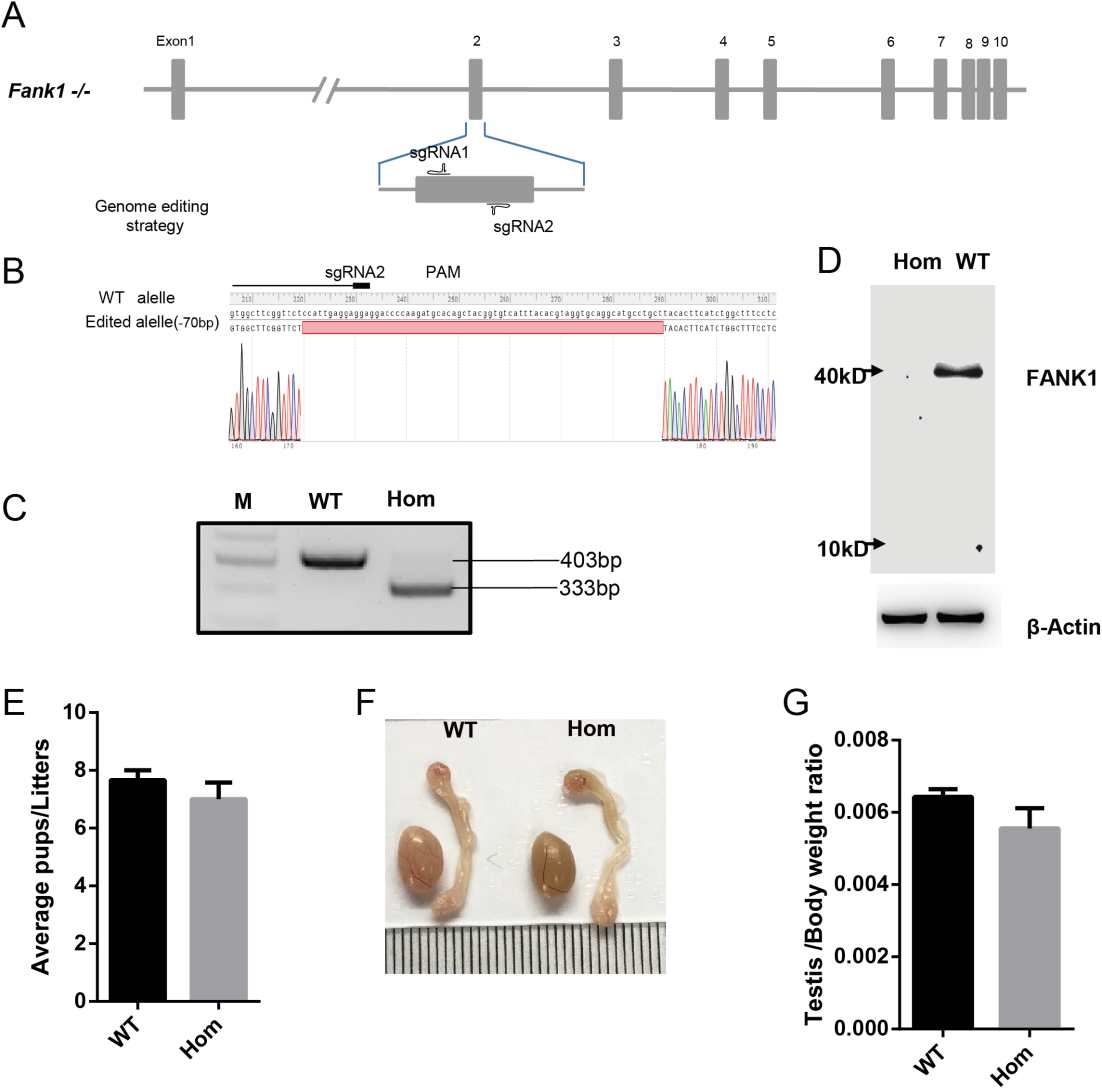
*Fank1*−/− mice are fertile. (A) Schematic diagram of CRISPR/Cas9 targeting strategy, the sgRNAs were designed on the basis of exon 2 of *Fank1*; (B) A 70-bp deletion of *Fank1* exon 2 was detected in *Fank1*−/− mice by Sanger sequencing and (C) agarose gel electrophoresis analysis; (D) FANK1 was not detected in *Fank1*−/− testis by western blot; (E) average pups per litter of wild-type and *Fank1*−/− mice, *n* = 3, *P* > 0.05; (F) testis and epididymis from wild-type and *Fank1*−/− adult mice; (G) average testis weight/body weight, *n* = 3, *P* > 0.05.

**Figure 3:**
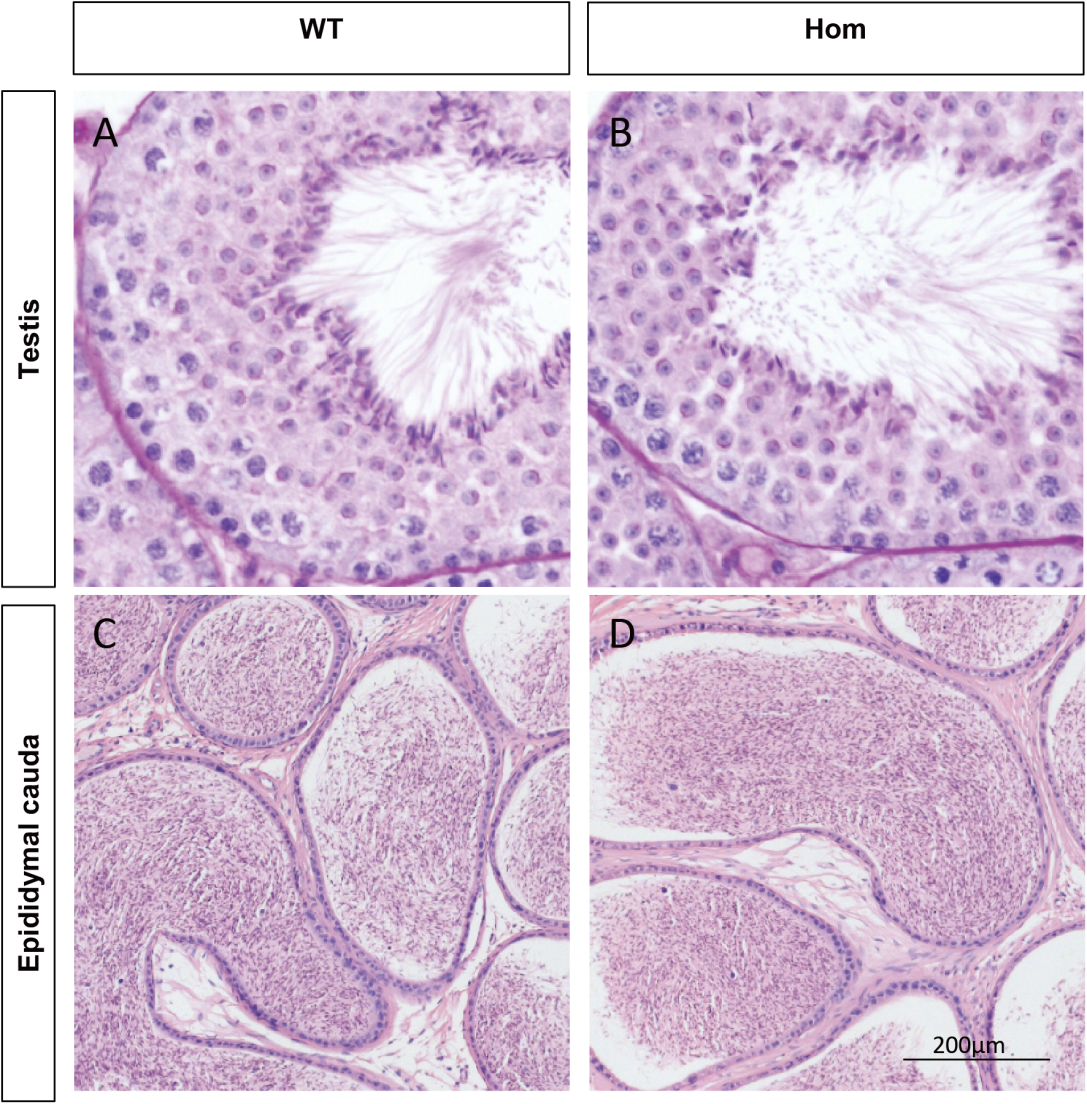
Spermatogenesis appears normal in *Fank1*−/− mice. Sections of periodic acid Schiff-stained testis from (A) wild-type and (B) *Fank1*−/− mice; Sections of hematoxylin and eosin-stained cauda epididymis from (C) wild-type and (D) *Fank1*−/− mice.

**Figure 4:**
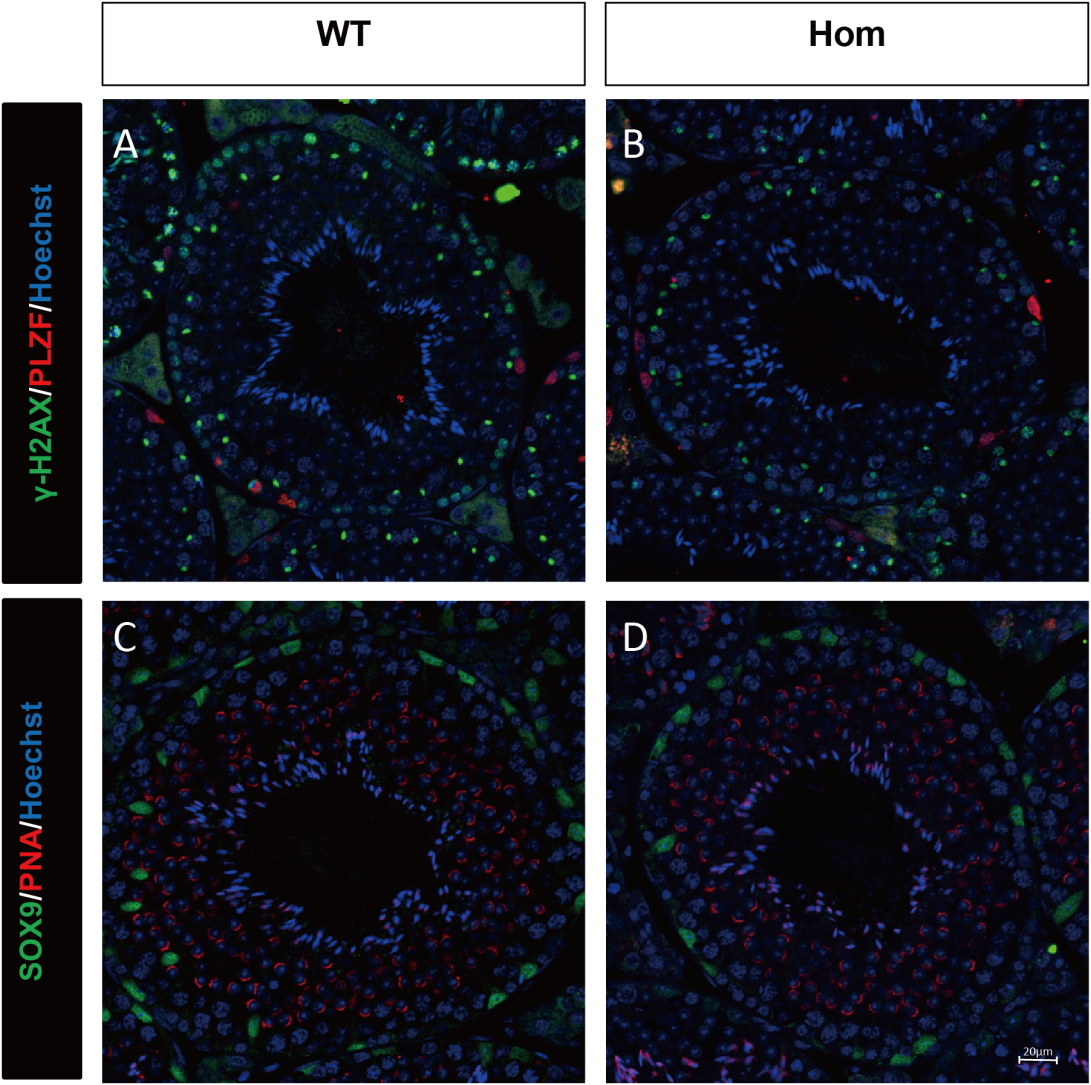
Spermatogenic markers appear normal in *Fank1*−/− mice. The spermatogonia (PLZF), spermatocytes (γ-H2AX) are comparable in testis sections from both (A) wild-type and (B) *Fank1*−/− mice; Spermatids (PNA) and Sertoli cells (Sox9) are comparable in testis sections from both (C) d-type and (D) *Fank1*−/− mice.

**Figure 5:**
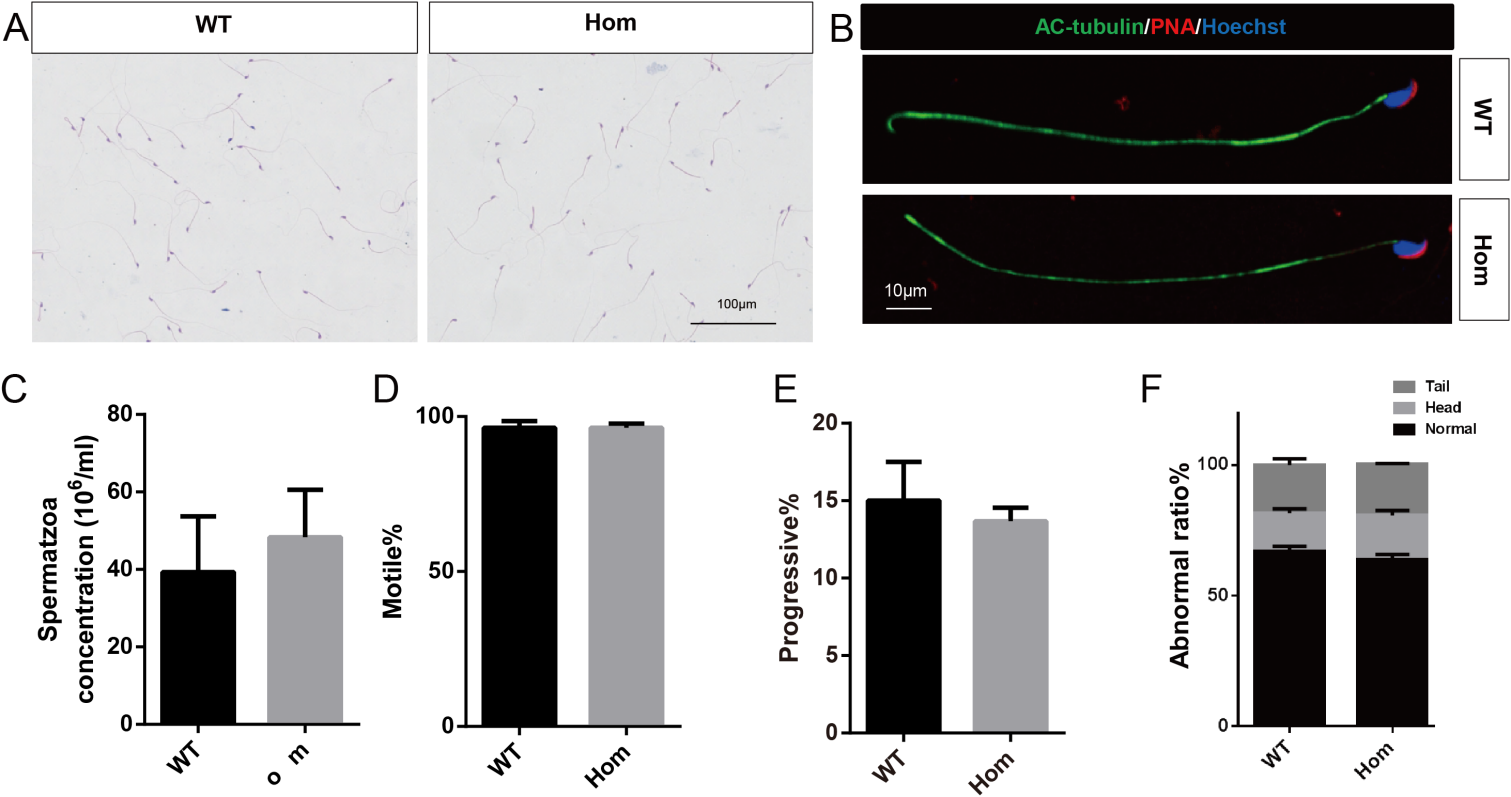
Spermatozoa appear normal in *Fank1*−/− mice. (A) Hematoxylin and eosin-stained spermatozoa from wild-type and *Fank1*−/− mice; (B) fluorescence detection of AC-tubulin, PNA from wild-type and *Fank1*−/− spermatozoa; (C) cauda epididymal sperm contents from wild-type and *Fank1*−/− mice, *n* = 3, *P* > 0.05; (D) average rate of motile sperm and (E) progressive sperm from wild-type and *Fank1*−/− mice, *n* = 3, *P* > 0.05; (F) abnormal epididymal sperm count from wild-type and *Fank1*−/− mice, *n* = 3, *P* < 0.05.

**Figure 6:**
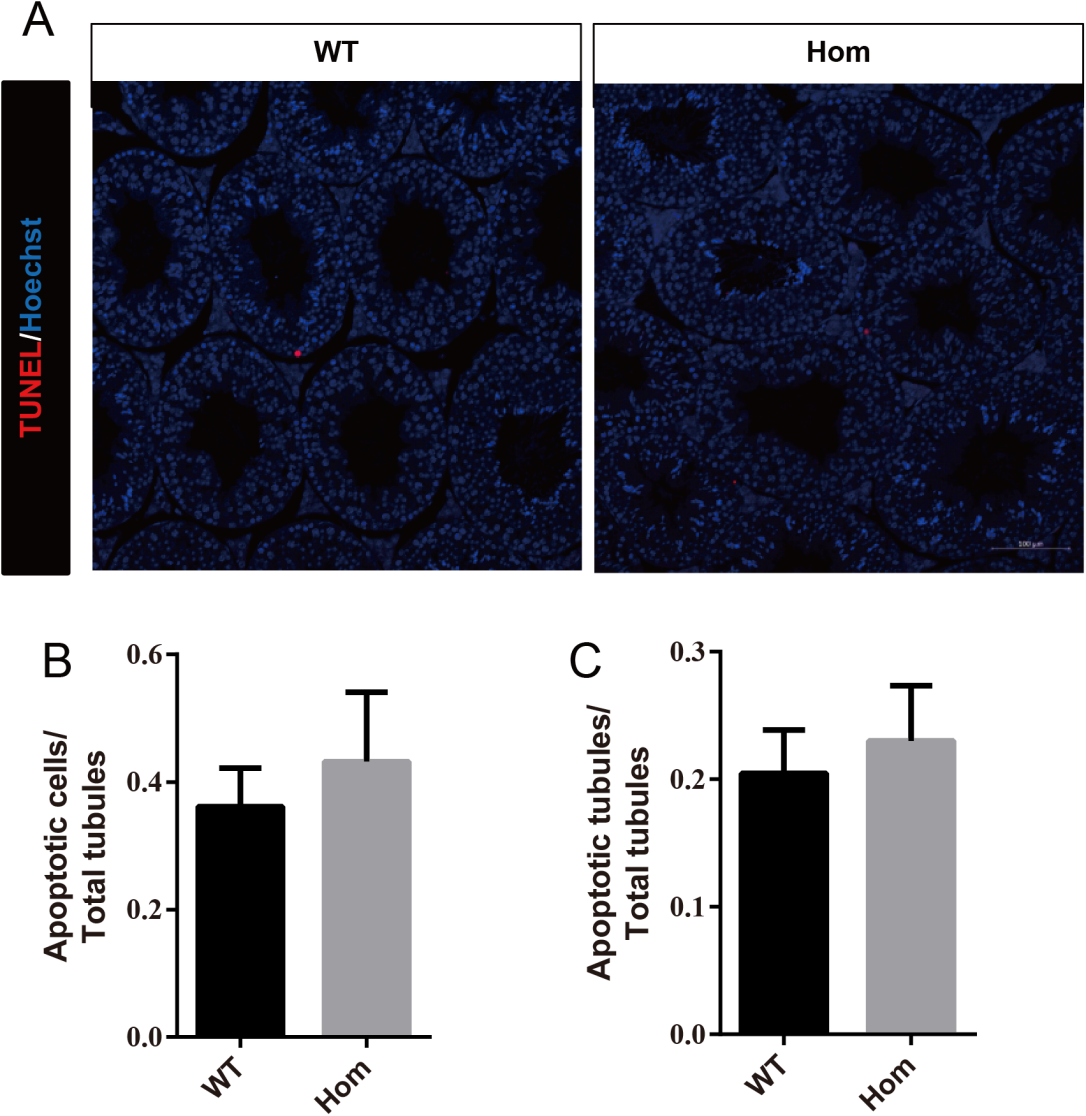
Apoptotic cells are not increased in *Fank1*−/− testes. (A) TUNEL assay of wild-type and *Fank1*−/− testes; (B) average apoptotic cells per seminiferous tubule; (C) average apoptotic cells per seminiferous tubules, *n* = 3, *P* > 0.05.

To confirm the in vivo function of *Fank1*, we generated *Fank1* mutant mice using the CRISPR/Cas9 system and a 70-bp deletion of exon 2 (Figs. 2A–2C). Neither FANK1 nor truncated FANK1 was detected in Fank1−/−-testis by western blot (Fig. 2D). *Fank1*−/− mice were viable and showed normal development. Intercrossing of *Fank1*+/− mice produced offspring of normal litter size at the predicted Mendelian and sex ratios. Similar to the *Fank1*-knockdown model, *Fank1*−/− males were fertile (Fig. 2E). In adult *Fank1*−/− mice, the testes and epididymis were similar in size to those of the wild-type mice (Figs. 2F and 2G). However, in contrast to the *Fank1*-knockdown model, histological analysis revealed the presence of spermatogenic cells in the seminiferous tubules of adult *Fank1*−/− mice (Figs. 3 and 4). Furthermore, compared with the wild-type mice, there were no significant differences in the morphology of *Fank1*−/− spermatozoa found in the cauda epididymides (Figs. 5A–5C). The whole epididymal sperm content and the average numbers of motile sperm were unaffected in homozygotic male mice (Figs. 5D–5F). TUNEL analysis of testicular sections revealed that both the number of apoptotic cells per tubule and the number of tubules containing apoptotic cells were unaffected in homozygotes (Fig. 6).

### Expression changes in Fank1−/− testis are not consistent with those of Fank1-knockdown mice

**Figure 7:**
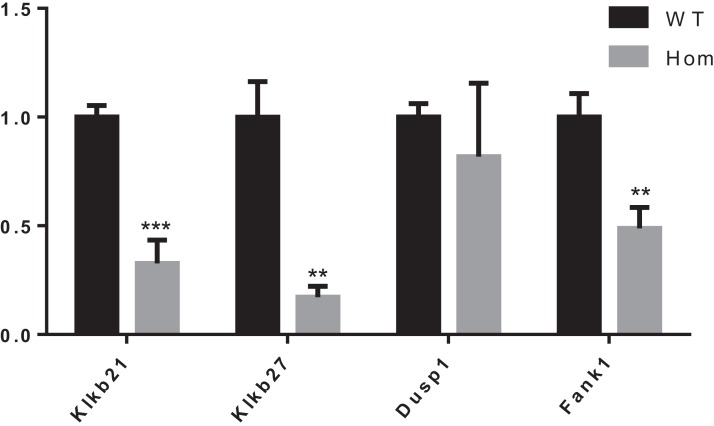
Expression changes in *Fank1*−/− testis. Quantitative RT-PCR analysis of *Dusp1*, *Klk1b21*, *Klk1b27* and *Fank1* in testis, *n* = 3, **P < 0.01; ***P < 0.001.

It was reported that Dusp1, Klk1b21 and Klk1b27 were overexpressed in *Fank1*-knockdown mice and may be direct targets of *Fank1* (Dong et al., 2014). However, in *Fank1*−/− testis, a reduction of Klk1b21 and Klk1b27 mRNA was detected but no increase in Dusp1 transcripts (Fig. 7). These results reveal differences in the molecular changes of *Fank1*-knockdown and *Fank1*-knockout mice.

## Discussion

In this study, we found that *Fank1* mRNA expression levels correlated negatively with the homozygous SNPs genotypes based on comparison with the GTEx database. This result showed the diversity of testicular *Fank1* expression levels in the population and prompted us to study *Fank1*-knockout mice. This phenomenon was not detected in studies of another testicular-specific gene *Pnldc1*, which is an evolutionarily conserved gene and essential for male fertility (Zhang et al., 2017). One explanation for this result may be that *Fank1* is dispensable for human reproduction. Thus, these genetic variants were retained during evolution.

Unlike shRNA-based *Fank1*-knockdown mice, we generated *Fank1* mutant mice using the CRISPR/Cas9 system. We found that the expression of *Fank1* was reduced by half in *Fank1* mutant mice (Fig. 7; Fig. S1), which is possibly caused by nonsense-mediated mRNA decay. Neither FANK1 nor truncated FANK1 was detected in *Fank1* mutant testis. Similar to the *Fank1*-knockdown model, *Fank1* mutant mice were fertile. Systematic studies have shown that neither testicular morphology nor sperm function is affected in *Fank1* mutant mice. In particular, the number of apoptotic cells were unaffected in *Fank1* mutant mice, while the number is markedly increased in *Fank1*-knockdown model mice (Dong et al., 2014). Although knockdown models are known to differ from knockout models, more in-depth studies are required to clarify these differences and their underlying mechanisms. The amino terminus of FANK1 contains a fibronectin type III domain and the carboxyl terminus includes five ankyrin repeats, which contain binding sites for DNA, heparin and the cell surface (Skorstengaard et al., 1986). Ankyrin repeats have been found in proteins of diverse function, such as transcriptional initiators and cell-cycle regulators (Skorstengaard et al., 1986). Lack of *Fank1* leads to a reduction in *Klk1b21* and *Klk1b27* transcripts via a mechanism that is unclear; however, transcriptional changes may also be induced as a compensatory mechanism, thus accounting for the absence of fertility changes in *Fank1*−/− males. In this study, we found no paralog of *Fank1* which may compensate for the *Fank1* mutation. Thus, we cannot explain the mechanisms underlying the phenotypic differences between the *Fank1* knockout and *Fank1* knockdown mouse models. Nevertheless, the *Fank1* knockout mouse model generated in this study provides a basic resource for studies of population genetics, and also expands our understanding of the differences in animal models established using different approaches.

## Conclusions

Although the diversity of testicular *Fank1* expression levels was detected in the population, no significant changes in epididymal sperm content and the number of apoptotic cells were observed in *Fank1*−/− male mice. In addition, a different pattern of Dusp1, Klk1b21 and Klk1b27 mRNA expression was detected in *Fank1*-knockout testis. These results reveal differences in the molecular changes between *Fank1*-knockdown mice and Fank1-knockout mice and provide a basic resource for population genetics studies.

## Supplemental Information

10.7717/peerj.6827/supp-1 Supplemental Information 1 The position of *Fank1* primer used for qPCR. *Fank1* primer pairs located in exon 5-7, and the gene editing location is exon 2 of *Fank1*.

10.7717/peerj.6827/supp-2 Supplemental Information 2 Table S1. SNPs associated with expression of the mRNAs of Fank1.

10.7717/peerj.6827/supp-3 Supplemental Information 3 Table S2. List of antibodies.

10.7717/peerj.6827/supp-4 Supplemental Information 4 Table S3. Primer sequences.

10.7717/peerj.6827/supp-5 Supplemental Information 5 Statistical documents.

10.7717/peerj.6827/supp-6 Supplemental Information 6 File S1. Raw data of Fig. 2B.

10.7717/peerj.6827/supp-7 Supplemental Information 7 File S2. Raw data of Fig. 2D.

10.7717/peerj.6827/supp-8 Supplemental Information 8 File S3. Raw data of Figs. 5C–5E.

10.7717/peerj.6827/supp-9 Supplemental Information 9 File S4. Raw data of Fig. 5F.

10.7717/peerj.6827/supp-10 Supplemental Information 10 File S5. Raw data of Fig. 6.

10.7717/peerj.6827/supp-11 Supplemental Information 11 File S6. Raw data of Fig. 7.

## Additional Information and Declarations

### Data Availability

The following information was supplied regarding data availability: The raw measurements are available in the Supplemental Files.
