# Supplementary figures and images for: Normal spermatogenesis in Fank1 (fibronectin type 3 and ankyrin repeat domains 1) mutant mice
Source: PeerJ. 2019 Apr 24;7:e6827. doi: 10.7717/peerj.6827 (PMC6486812; doi:10.7717/peerj.6827)

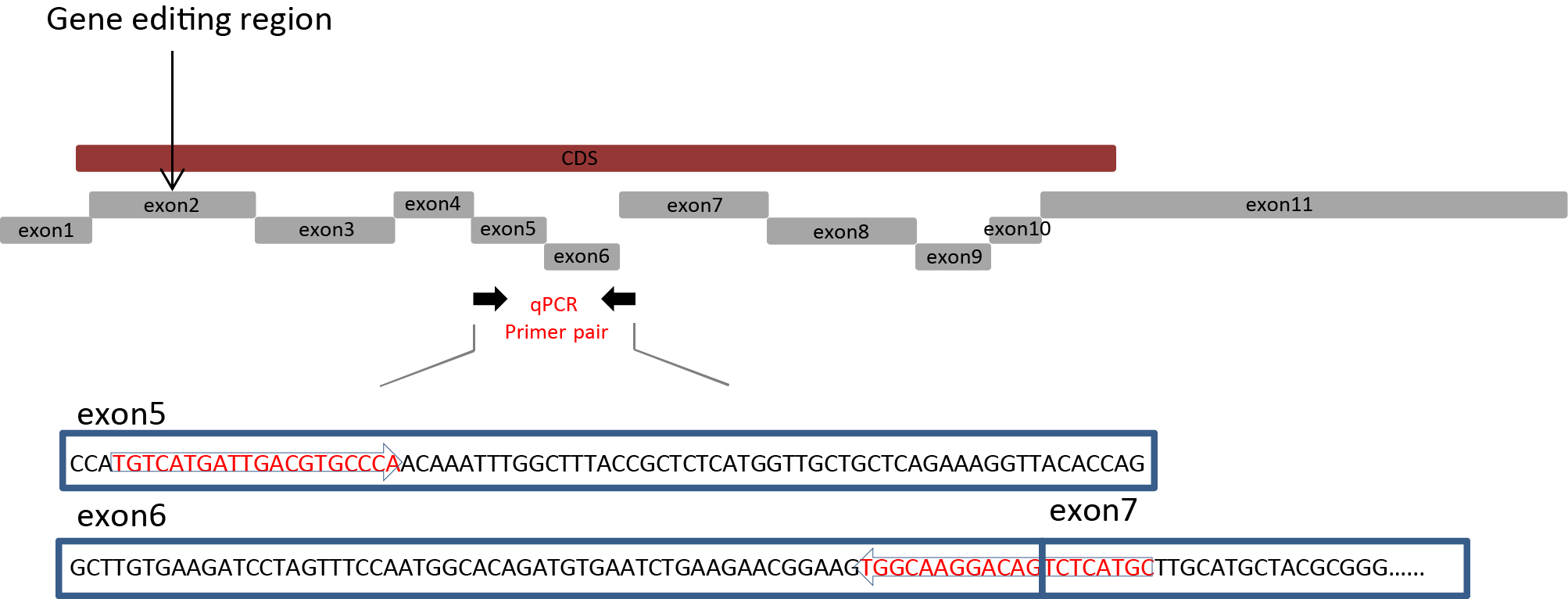

Supplement: Supplemental Information 1 — Fank1 primer pairs located in exon 5-7, and the gene editing location is exon 2 of Fank1. [file peerj-07-6827-s001.png]
